# Endothelial progenitor cells-derived exosomes transfer microRNA-30e-5p to regulate Erastin-induced ferroptosis in human umbilical vein endothelial cells via the specificity protein 1/adenosine monophosphate-activated protein kinase axis

**DOI:** 10.1080/21655979.2022.2025519

**Published:** 2022-01-22

**Authors:** Jia Xia, Xiaoying Song, Jing Meng, Danfei Lou

**Affiliations:** a Department of Rheumatology, Shanghai Municipal Hospital of Traditional Chinese Medicine, Shanghai University of Traditional Chinese Medicine, Shanghai, China; b Department of Geriatrics, Shanghai Municipal Hospital of Traditional Chinese Medicine, Shanghai University of Traditional Chinese Medicine, Shanghai, China

**Keywords:** Endothelial progenitor cells, exosomes, human umbilical vein endothelial cell, miR-30e-5p, SP1, ferroptosis, endothelial injury, angiogenesis

## Abstract

Ferroptosis is a kind of cell death triggered by intracellular phospholipid peroxidation. Human umbilical vein blood endothelial progenitor cells-Exosomes (EPCs-Exos) affect ferroptosis. This study sought to explore the mechanism of EPCs-Exos in human umbilical vein endothelial cell (HUVEC) ferroptosis. EPCs-Exos were isolated and identified. HUVECs were treated with Erastin at IC50 concentration. Ferroptosis-related indexes and iron ion content were detected using kits. HUVEC migration and angiogenesis before/after ferroptosis inhibitor treatment were observed by cell scratch and angiogenesis assays. After Erastin induction, HUVECs were transfected with miR-30e-5p mimic, or treated with EPCs-Exos and EPCs-Exos transfected with miR-30e-5p inhibitor. miR-30e-5p expression was detected by RT-qPCR. The binding relationship between miR-30e-5p and specificity protein 1 (SP1) was verified by dual-luciferase assay. SP1 expression was detected by Western blot. HUVECs treated with Erastin and EPCs-Exos were transfected with pcDNA3.1-SP1. Protein levels of adenosine monophosphate-activated protein kinase (AMPK) and p-AMPK were detected by Western blot. EPCs-Exos inhibited Erastin-induced HUVEC ferroptosis and endothelial injury. Erastin inhibited miR-30e-5p and EPCs-Exo treatment recovered miR-30e-5p expression. miR-30e-5p was encapsulated in EPCs-Exos. After inhibiting miR-30e-5p in EPCs, the inhibitory effect of EPCs-Exos on HUVEC ferroptosis was attenuated. miR-30e-5p targeted SP1. Overexpression of SP1 partially reversed the effect of EPCs-Exos on improving HUVEC ferroptosis and increasing phosphorylation levels of AMPK. Collectively, EPCs-Exos inhibited Erastin-induced HUVEC ferroptosis by upregulating miR-30e-5p, inhibiting SP1, and activating the AMPK pathway.

## Introduction

Endothelial cells are the innermost layer of the flat epithelium of the heart, lymphatic vessels, and blood vessels, which can secrete vasoactive cytokines, regulate vascular tone, maintain the permeability of blood vessels, and provide a smooth surface for the blood flow [1]. Endothelial injury is closely associated with the development of multiple cardiovascular and cerebrovascular diseases [2]. However, the mechanism of vascular endothelial injury is not been fully understood so far. Ferroptosis is a unique type of non-apoptotic and iron-dependent mode of cell death that is characterized by excess accumulation of lipid-reactive oxygen species (ROS), which is different from autophagy, necrosis, and apoptosis in the cell function and morphology [3]. Ferroptosis is induced by the iron-catalyzed lipid peroxidation initiated by the enzymatic and nonenzymatic mechanisms [4]. It is implicated in multiple diseases including tumors, tissue injuries, and ischemia diseases [5]. There is evidence to suggest that the inhibition of ferroptosis can attenuate endothelial dysfunction [6]. Therefore, inhibition of ferroptosis may be a promising target for the management of endothelial injury in cardiovascular diseases.

Endothelial progenitor cells (EPCs) are regarded as an essential contributor to the repair of endogenous vascular through involvement in the endothelial regeneration [7]. Exosomes (Exos) are a kind of extracellular vesicles, which are identified to be an important way of communication between organ to organ and cells to cells by delivering their cargoes, including microRNAs (miRNAs), mRNAs, and proteins [8]. EPCs-derived exosomes (EPCs-Exos) have been proposed to get involved in the physiological and pathological mechanisms of diverse diseases and have a protective effect on endothelial cells [9]. Notably, an existing study identified the regulation of Exos in cell ferroptosis in the irradiated fibroblasts [10]. miRNAs are well-known small noncoding RNAs, which have essential effects on the regulation of post-transcriptional genes and regulate their downstream target genes through mRNA destabilization and translational inhibition in cells [11]. Moreover, miRNAs can relieve various diseases induced by ferroptosis [12]. Endogenous miR-30e-5p in endothelial cells can regulate mRNAs encoded by genes relevant to hypertension [13]. Lipopolysaccharide reduced miR-30e-5p expression in human brain microvascular endothelial cells [14]. However, whether EPCs-Exos can protect HUVECs from ferroptosis and endothelial injury through the miR-30e-5p remains to be studied. In light of the aforementioned literature, we speculated that HUVECs could improve the ferroptosis of HUVECs by uptaking EPCs-Exos. The current study sought to investigate the molecular mechanism of EPCs-Exos in regulating HUVECs ferroptosis, so as to find a new target for the treatment of endothelial cell ferroptosis-related diseases.

## Materials and methods

### Ethics statement

This study was conducted with approval of the academic ethics committee of Shanghai municipal Hospital of Traditional Chinese Medicine. All procedures strictly followed the code of Declaration of Helsinki.

### *EPC culture and identification* [15,16]

Human umbilical vein blood-derived EPCs (HEPC, Cat No. CP-H164, Procell, Wuhan, Hubei, China) were washed twice with phosphate buffer saline (PBS) and centrifuged at 100 g. The cells were suspended in the endothelial basal growth medium supplemented with EGM-2 mV SingleQuots and 5% heat-inactivated fetal bovine serum (FBS) (Lonza, Basel, Switzerland). Then, the solution was spread into 24-well plates coated with 10 μg/mL human plasma fibronectin (FN, Millipore, Billerica, MA, USA). After 96 h of removal of non-adherent cells, the non-adherent cells were cultured in the above medium, and spindled cells were seen after 7 days. Next, the colonies of endothelial-like cells were cultured to confluence, trypsinized, and evenly spread on the new 24-well plates as the first generation. The medium was refreshed every 3 days. When attaining 80% confluence, the cells were detached with 0.25% trypsin and passaged at the ratio of 1:2. The EPCs of the 3^rd^-6^th^ generation were used for the following experiments.

The cells were cultured with 1,1′-dioctadecyl-3,3,3′,3′-tetramethylindocarbocyanine perchlorate-acetylated-low density lipoprotein (Dil-Ac-LDL) (15 mg/L) and fluorescein isothiocyanate Ulex Europaeus agglutinin-I (FITC-UEA-I) (10 mg/L) for 14 d. The spindled cells stained with Dil-Ac-LDL and FITC-UEA-I were observed under an inverted fluorescence microscope. The expressions of adherent EPC surface proteins CD31 (1:1000, ab9498, Abcam Cambridge, MA, USA) and CD34 (1:1000, ab54208, Abcam) were determined using immunofluorescence staining. The cell staining results were observed using a fluorescence microscope (Zeiss, LSM 700B, Germany).

### EPCs-Exo extraction and identification[16]

When the EPCs reached 80% confluence, the original medium was removed, followed by treatment with FBS-free EGM-2 MV (endothelial growth medium, Cambrex-Clonetics, Walkersville, MD, USA) for 1 day. The medium was placed into a 1.5 mL eppendorf (EP) tube and centrifuged at 2000 g at 4°C for 30 min. The supernatant was placed in a new 1.5 mL EP tube and the precipitate was discarded. The supernatant was incubated overnight with the 1/2 volume Exo separation kit (Thermo Fisher Scientific, Waltham, MA, USA) at 4°C. The next day, the EP tube was centrifuged at 10,000 g at 4°C for 1 h. The precipitate was suspended with sterilized phosphate buffer saline (PBS). The EPCs-Exo suspension was stored at −80°C. After treatment with FBS-free EGM-2 MV for 1 day, EPCs were co-cultured with 5 μM specific inhibitor GW4869 (Sigma-Aldrich, St. Louis, MO, USA) for 48 h to block the release of Exos, as the control. The protein content of EPCs-Exos was determined using the bicinchoninic acid kit (BCA, Thermo Fisher Scientific).

The morphology of EPCs-Exos was observed using a transmission electron microscope (TEM). The Exos showed a complete double-layer vesicle structure with a diameter of 30–200 nm. The Exos were rinsed with PBS 3 times and fixed with 1% glutaraldehyde for 6 min. The 10 *μ*L of each sample was placed into a copper mesh and precipitated for 3 min. The remaining liquid was pipetted carefully from the edge of the filter paper. After that, the filter paper was rinsed with PBS. The samples were negatively stained with phosphotungstic acid and dried at room temperature for 2 min and imaging, and then observed under a TEM at 80 kV. The images were obtained. The size distribution and concentration of EPCs-Exos were examined using Nanosizer™ technology (Malvern Instruments, Malvern, Worcestershire, UK). Afterward, 10 μL Exos and 990 μL 0.22 μm filtered sterile PBS were slowly pushed using a 1 mL syringe and irradiated with laser. The movement was recorded using a 30-second sample video and analyzed using nanoparticle tracking analysis (NTA) software. The expressions of Exo-specific surface proteins CD9, CD63, CD81, and Calnaxin were determined using Western blot (WB). The cell supernatant supplemented with GW4869 was used as the control. Exos derived from EPCs and the EPCs transfected with inhibitor-NC and miR-30e-5p inhibitor were set as Exos, Exos^inhibi-NC^ and Exos^miR-inhibi^.

### Cell uptake Exo assay

The EPCs-Exos were labeled using PKH67 kits (Sigma-Aldrich). Exos were incubated with PKH67 membrane dye (4 μL, Sigma-Aldrich) and diluent C (1 mL) for 4 min. The labeled Exos were filtered using the Exoquick Exo precipitation solution and suspended in the basic medium. HUVECs were incubated with the above solution (250 μL) for 3 h. Then, the samples were incubated with 4% paraformaldehyde (1 mL) for 0.5 h. The nucleus was stained with 4’,6-diamidino-2-phenylindole (DAPI). The images were observed using a fluorescence microscope (Zeiss, LSM700B, Germany).

### HUVEC culture and treatment

HUVECs (ATCC) were cultured in the EGM-2 medium (Lonza, Walkersville, MD, USA) and cell concentration was adjusted to 2 × 10^4^ cells/well. The samples were seeded in 6-well plates, added with gradient concentrations of ferroptosis inducer Erastin (0 μM, 2.5 μM, 5 μM, 7.5 μM, and 10 μM) or ferroptosis inhibitor Fer-1 (0.5 μM) [5], and cultured in an incubator (Nuare, USA) containing 5% CO_2_ at 37°C for 24 h. The cells were allocated as the following groups: HUVECs group (HUVECs without any treatment), DMSO group (HUVECs treated with Erastin solvent dimethyl sulfoxide (DMSO)), Erastin group (HUVECs treated with 5 μM Erastin), Erastin + Fer-1 group (HUVECs treated with 5 μM Erastin and 0.5 μM Fer-1), Erastin + EPCs-Exos group (HUVECs treated with Erastin after co-culture with EPCs-Exos), Erastin + mimic NC group (after transfection with mimic NC, HUVECs were treated with Erastin), Erastin + miR-30e-5p mimic group (after transfection with miR-30e-5p mimic, HUVECs were treated with Erastin), Erastin + EPCs-Exos^inhibi-NC^ group (HUVECs treated with Erastin after co-cultured with EPCs-Exos transfected with miR-30e-5p inhibitor), Erastin + EPCs-Exos + pc3.1-NC group (HUVECs treated with Erastin after co-culture with EPCs-Exos transfected with pcDNA3.1-NC), and Erastin + EPCs-Exos + pc3.1-SP1 group (HUVECs treated with Erastin after co-cultured with EPCs-Exos transfected with pcDNA3.1-specificity protein 1 (SP1)).

### Cell transfection

Inhibitor NC, miR-30e-5p inhibitor, mimic NC, miR-30e-5p mimic, pcDNA3.1-NC, and pcDNA3.1-SP1 (Shanghai Genechem Co., Ltd., Shanghai, China) (miRNA-inhibitor/NC 50 nM, miRNA-mimic/NC 40 nM, pcDNA3.1-SP1/NC 40 nM) were respectively transfected into experimental cells or 293 T cells using Lipofectamine 2000 (11,668–019, Invitrogen, Carlsbad, CA, USA) according to the instructions. miR-30e-5p inhibitor is a specially modified miRNA inhibitor synthesized by chemical synthesis, which can specifically bind with mature miR-30e-5p to inhibit its expression; pcDNA3.1-SP1 is a plasmid carrying SP1 sequence, which can stably express SP1 in cells. After 48 h of transfection, the following experiments were performed.

### 3-(4,5-dimethylthiazol-2-yl)-2,5-diphenyltetrazolium bromide (MTT) assay

The cells with different treatment protocols were seeded in 96-well plates at the density of 8 × 10^3^ cells/well, added with 20 μL prepared MTT solution in each well, and cultured at 37°C for 4 h. After culture according to the requirements of the following experiments, the cells were added with 200 μL DMSO and shaken for 10 min with the procedure set by a microplate reader for complete dissolution and crystallization. The optical density (OD) value was measured at 450 nm.

### Lipid-ROS detection[17]

The concentration of cells was adjusted to 1 × 10^6^ cells/well and cells were seeded in 6-well plates overnight. Then, the cells were treated using the BODIPY™581/591 C11 method (D3861, Invitrogen) for the detection of lipid-ROS. The cell fluorescence was immediately detected using an Accuri C6 flow cytometer (BD Biosciences, San Jose, CA, USA). The cell experiment was conducted 3 times independently. The data were analyzed using FlowJo software (version 10.0, FlowJo LLC, USA).

### Malondialdehyde (MDA) and glutathione (GSH) determination

The levels of MDA and GSH in HUVECs were determined using corresponding kits (Abcam) according to the provided instructions. The contents of GSH and MDA in HUVECs were determined using the colorimetry method. GSH or MDA in the sample was reacted with the kits to determine the absorbance of the sample. The contents of GSH and MDA were determined by comparing with the standard.

### Iron ion determination

The total iron content in HUVEC lysate was determined using an iron ion detection kit (Abcam). HUVECs or HUVECs treated with Exos were homogenized with normal saline and PBS and centrifuged at 16,000 × g for 10 min for removal of insoluble matter. Then, 50 µL samples were added with 5 µL iron reductant to obtain total iron content (Fe ^3+^ and Fe ^2+^). Subsequently, the samples were added with 100 μL iron probe solution and incubated at 25°C in the dark conditions for 60 min. The absorbance at 593 nm was detected using the spectrophotometry method.

### Cell scratch assay

The cells were seeded in 6-well plates at 5 × 10^5^ cells/well and cultured to confluence. After the cells were treated using the above method, the center of the cell layer of each well was scratched using a 1000 μL sterile micropipette tip. Then, the cells were washed twice with PBS and cultured in the FBS-free medium. After 0 h and 24 h of the scratch, 3 independent field images of each group were randomly collected under a bright field microscope (Olympus). The scratch width was quantitatively measured using ImageJ software (v1.52a, NIH, Bethesda, MD, USA) and the mobility was calculated.

### Angiogenesis assay

The 100 μL liquefied Matrigel (Becton Dickinson Company) was added to the 24-well plates and placed at 37°C until the Matrigel solidified. Then, the cells were seeded in 24-well plates. The angiogenesis of HUVECs was observed using a bright-field microscope after 8 h. The branch number and length of vessels were quantified using ImageJ software (v1.52a, National Institutes of Health).

### Reverse transcription quantitative polymerase chain reaction (RT-qPCR)

TRIzol (Invitrogen) was used to lysate HUVECs. The RNA extraction kit (Tiangen Biotech Co., Ltd.) was used to extract total RNA. The SuperScript IV kit (Thermo Fisher Scientific) was used for reverse transcription according to the provided instructions. cDNA was synthetized at 50°C for 10 min and at 80°C for 10 min. The qPCR was performed according to the PowerUp™ SYBR™ Green Master mix (Thermo Fisher Scientific, Inc.) and ABI ViiA 7 System (Thermo Fisher Scientific, Inc.) under the conditions: pre-denaturation at 95°C for 120 s and 40 cycles of denaturation at 95°C for 15s, and extending at 60°C for 60s. The 2^−ΔΔCT^ method was used for quantification [18]. U6 was used as the internal reference for miR-30e-5p and GAPDH was used as the internal reference for SP1. The primer sequences are shown in Table 1.

**Table 1.** Primer sequence

Name of primer	Sequences
miR-30e-5p-F	TGTAAACATCCTTGACTGGAAG
miR-30e-5p-R	GCGAGCACAGAATTAATACGAC
SP1-F	ATGAAATGA CAGCTGTGGT GA
SP1-R	TGAA AAAGGAGTTG GTGGCAA
GAPDH-F	CAAGCAACTGTCCCTGAG
GAPDH-R	TAGACAGAAGGTGGCACA
U6-F	ATTGGAACGATACAGAGAAG
U6-R	GGAACGCTTCACGAATTTG

### RNase protection assay

RNase treatment was conducted to confirm that miR-30e-5p was encapsulated in Exos or bound to the Exo surface. Exos were resuspended with PBS, added with 2 μg/μL RNAse (Purelink RNase A, Life technologies), and incubated at 37°C for 20 min. Subsequently, the Exos were treated with 0.1% TritonX-100 for 20 min to destroy the integrity of Exo membrane structure, followed by RNase treatment. After that, Exos were added with the lysis buffer to inhibit the reaction and isolate RNA. The relative expression of miR-30e-5p was determined.

### Dual-luciferase assay

The target binding sites between miR-30e-5p and SP1 were analyzed using the Starbase website (http://starbase.sysu.edu.cn). The wild-type (SP1-WT) and mutant (SP1-MUT) luciferase plasmids were constructed by cloning the binding sequence and mutant sequence into the luciferase vector pGL3 (Pro-mega, Madison, WI, USA), respectively. The 293 T cells (ATCC) were seeded in 6-well plates (5 × 10^5^ cells/well). After 24 h of incubation, the 293 T cells were transfected with constructed luciferase vectors and mimic NC or miR-30e-5p mimic (Shanghai Genechem Co., Ltd.) (miRNA-mimic 30 nM) according to the instructions of Lipofectamine 2000 (11,668–019, Invitrogen). After 24 h, the luciferase activity was evaluated using Dual-Lucy Assay kit (Solarbio, Beijing, China). The cell experiment was conducted 3 times independently.

### Western blot (WB)

Total protein was extracted from cells using the radio-immunoprecipitation assay (RIPA) lysis buffer (Beyotime, Shanghai, China). The whole-cell protein extract was identified using the BCA method. The same amount of protein lysates was isolated using sodium dodecyl sulfate polyacrylamide gel electrophoresis (SDS-PAGE) on 12% polyacrylamide gel (Invitrogen) and transferred to nitrocellulose membrane membranes (Bio-Rad, Hercules, CA, USA). After blocking with 5% skim milk in tris buffered saline-Tween20, the samples were rinsed once and incubated with primary antibodies at 4°C overnight. GAPDH was used as the loading control. The samples were incubated with coupling horseradish peroxidase IgG secondary antibody (1:1000, ab150077, Abcam), and the band was visualized using enhanced chemiluminescence (ECL) (Amersham Biosciences). Primary antibodies were CD9 (1:1000, ab92726, Abcam), CD63 (1:1000, ab134045, Abcam), CD81 (1:1000, ab79559, Abcam), Calnaxin (1:1000, ab92356, Abcam), SP1 (1:1000, ab124804, Abcam), p-adenosine monophosphate-activated protein kinase (AMPK) (1:1000, ab133448, Abcam), AMPK (1:1000, ab32047, Abcam) and GAPDH (1:1000, ab9485, Abcam).

### Statistical analysis

SPSS 21.0 statistical software (IBM Corp. Armonk, NY, USA) and GraphPad Prism 8.0 software (GraphPad Software Inc San Diego, CA, USA) were used for data analysis and mapping. The experimental data were expressed as mean ± standard deviation. Independent *t* test was used for comparisons between 2 groups. One-way analysis of variance (ANOVA) was used for comparisons among multi-groups. Tukey’s multiple comparisons test was used for the post hoc test. *P* value was obtained by a bilateral test. In all statistical references, a value of *P*< 0.05 was indicative of statistical significance.

## Results

Ferroptosis can cause vascular endothelial cell dysfunction (6). EPCs are an important factor involved in vascular endothelial regeneration and endogenous vascular function repair (7). EPCs-Exos can transmit cytokines through a variety of ways to protect endothelial cells (8, 9). Therefore, we speculated that EPCs-Exos could affect the ferroptosis of endothelial cells by transmitting miRNA. We isolated and identified EPCs-Exos and co-cultured them with Erastin-induced HUVECs to observe the changes in the cellular biological function of HUVECs, and further explored the specific molecular regulatory mechanism of EPCs-Exos in regulating the changes of endothelial cell function.

### EPCs-Exos could be internalized by HUVECs

**Figure 1.**
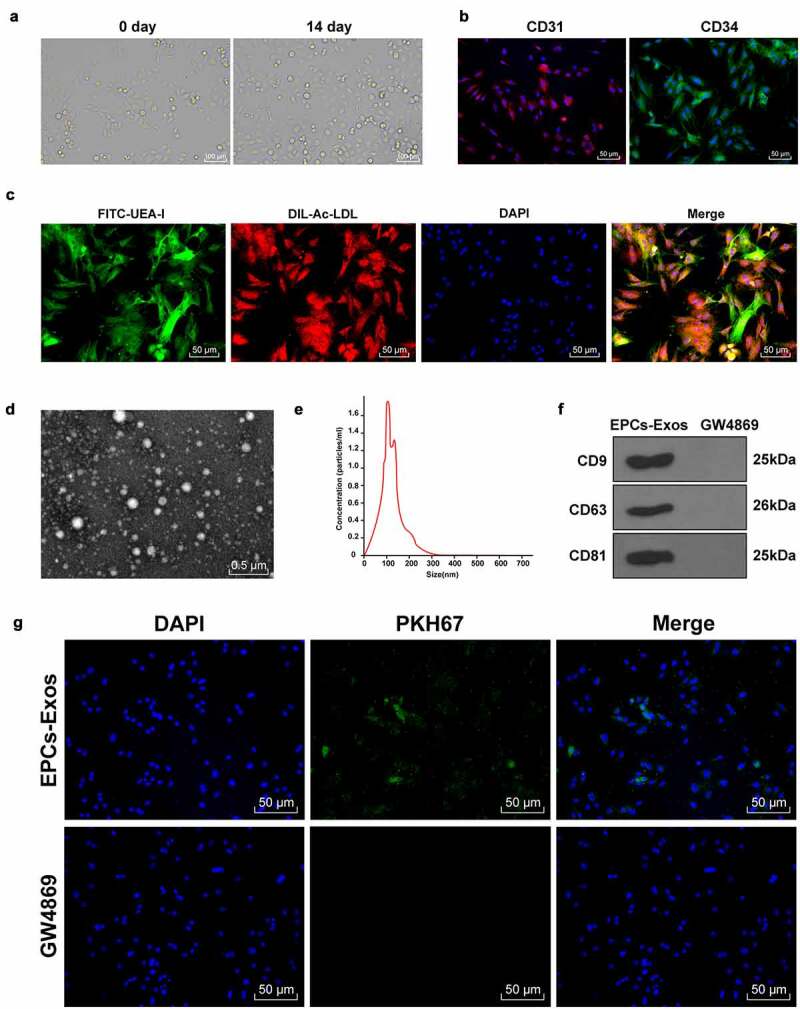
EPCs-Exos could be transferred to HUVECs. EPCs-Exos were extracted and identified. The uptake of EPCs-Exos by HUVECs was observed. (a) Morphological changes of EPCs were observed using an inverted microscope; (b) EPCs markers CD31 and CD34 were detected by immunofluorescence; (c) FITC-UEA-I and Dil-ac-LDL were detected by immunofluorescence; (d) The ultrastructure of Exos were observed using a TEM; (e) The diameter distribution and concentration of Exos were determined by NTA; (f) Exos mark proteins CD9, CD63, CD81, and Calnaxin were detected by WB; (g) The entry of PKH67-labeled Exos into HUVECs was detected by immunofluorescence. The cell experiments were repeated 3 times independently.

When EPCs were cultured for 14 days, EPC volume was increased and appeared as paving stones (Figure 1(a)); the fluorescence microscope showed positive expression of CD31 and CD34 in EPCs, and the positive cell rates were 98.17% and 96.83%, respectively (Figure 1(b)). The function of EPCs was identified by FITC-UEA-I and Dil-ac-LDL staining (Figure 1(c)). Red fluorescence was observed in the cytoplasm of EPCs under a fluorescence microscope, indicating that EPCs could absorb Dil-ac-LDL; green fluorescence was observed in EPCs membrane, indicating that EPCs could bind to FITC-UEA-I; the cells showing double fluorescence staining were the differentiating EPCs [19]. The Exos purified by ultracentrifugation were stained with 3% phosphotungstic acid. The morphology and particle size of Exos were observed by TEM and NTA analysis (Figure 1(d,e)). Our findings denoted that the size of the Exo was in accordance with the general characteristics of Exos. WB detected the expressions of specific marker proteins CD9, CD63, and CD81 in Exos (Figure 1(f)). The results above suggested that EPCs-Exos were successfully isolated. EPCs-Exos can improve the dysfunction of HUVECs [20]. To further study the effect of EPCs-Exos on HUVECs, EPCs-Exos labeled by PKH67 were co-cultured with HUVECs and the uptake of EPCs-Exos by HUVECs was observed after 12 h (Figure 1(g)). It was observed that a large number of EPCs-Exos entered HUVECs and were distributed around the nucleus. These results suggested that EPCs could transfer Exos into HUVECs.

### Erastin induced ferroptosis and endothelial injury of HUVECs

**Figure 2.**
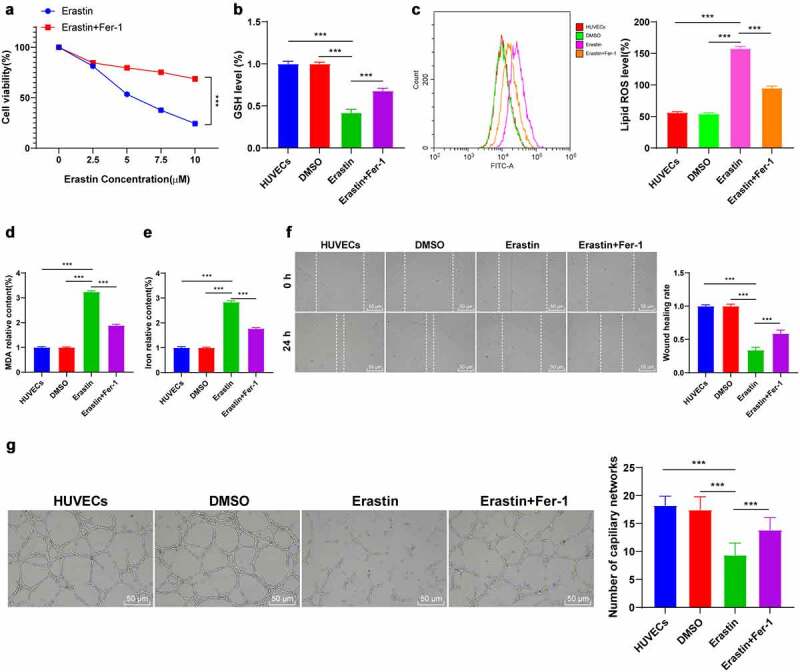
The endothelial function of HUVECs was impaired by Erastin-induced ferroptosis. After Erastin treatment, HUVECs were added with ferroptosis inhibitor Fer-1, and the changes of HUVECs were observed. (a) The effect of Erastin of gradient concentrations on HUVECs was detected by MTT assay; (b) GSH content determination; (c) Lipid-ROS content determination; (d) MDA content determination; (e) Iron ion content determination; (f) Cell migration was detected by scratch assay; (g) Cell angiogenesis was detected by angiogenesis assay. The cell experiments were repeated 3 times independently. The data in the figure were all measurement data and were expressed as mean ± standard deviation. One-way ANOVA was used for data analysis. Tukey’s multiple comparisons test was used for the post hoc test. *** *P* < 0.001.

Ferroptosis and endothelial injury of HUVECs were induced by Erastin. MTT demonstrated that the half inhibitory concentration IC50 of Erastin was 5 μM and the inhibitory effect of IC50 concentration Erastin on cell viability was significantly reversed by 0.5 μM ferroptosis inhibitor (Figure 2(a)). Afterward, HUVECs were treated with Erastin of IC50 concentration. The ferroptosis-related indexes were detected using the corresponding reagent or kit. Our findings denoted that the content of GSH was reduced, lipid-ROS and MDA were significantly increased, and iron ions were increased by about 2.5 times (all *P* < 0.001, Figure 2(b-e)). Finally, cell migration and angiogenesis were detected by cell scratch assay and angiogenesis assay (Figure 2(f,g)). It was revealed that HUVEC migration and angiogenesis were decreased after Erastin-induction (all *P* < 0.001), while the changes of cell functions were partially improved after adding with ferroptosis inhibitor Fer-1 (Figure B-G, all *P* < 0.001). These results suggested that Erastin-induced ferroptosis impaired the endothelial functions of HUVECs.

### EPCs-Exos improved ferroptosis and endothelial injury of HUVECs through miR-30e-5p

**Figure 3.**
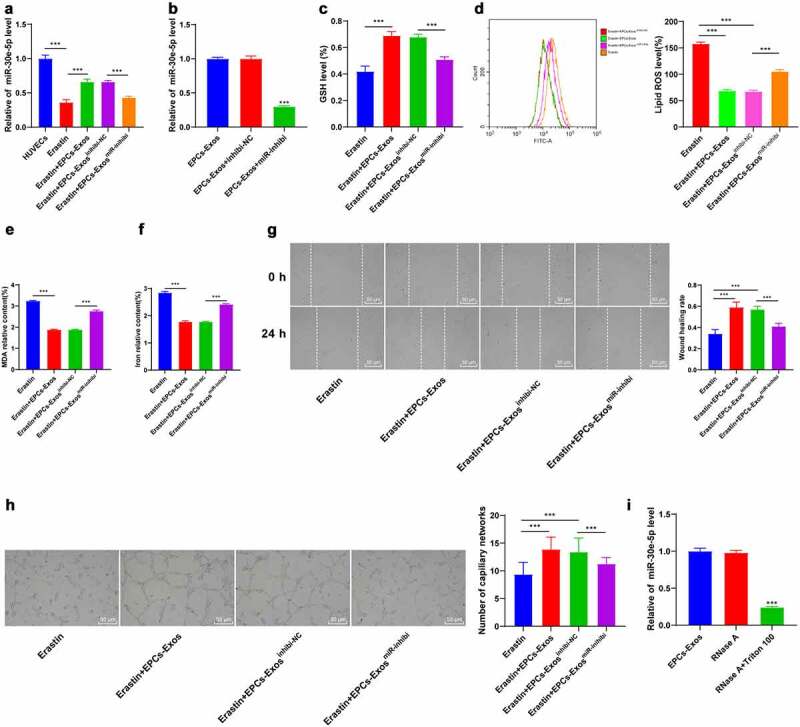
EPCs-Exos improved HUVECs ferroptosis and endothelial injury through regulating miR-30e-5p in HUVECs. HUVECs treated with Erastin were co-cultured with EPCs-Exos, EPCs-Exos^inhibit-NC^, or EPCs-Exos^miR-inhibi^, and the changes of HUVEC ferroptosis and injury were observed. (a) The expression of miR-30e-5p in HUVECs of each group was detected by RT-qPCR; (b) The expression of miR-30e-5p in EPCs was detected by RT-qPCR; (c) GSH content determination; (d) Lipid-ROS content determination; (e) MDA content determination; (f) Iron ion content determination; (g) Cell migration was detected by scratch assay; (h) Cell angiogenesis detection; (i) The expression of miR-30e-5p was detected by RT-qPCR after Exos were treated with RNase and detergent. The cell experiments were repeated 3 times independently. The data in the figure were all measurement data and were expressed as mean ± standard deviation. One-way ANOVA was used for data analysis. Tukey’s multiple comparisons test was used for the post hoc test. *** *P* < 0.001.

miR-30e-5p is associated with endothelial cell injury [14]. RT-qPCR demonstrated that after Erastin induction, the expression pattern of miR-30e-5p in HUVECs was downregulated, while the miR-30e-5p expression was partially recovered after the co-culture of EPCs-Exos and HUVECs (*P* < 0.001, Figure 3(a)). We found that miR-30e-5p existed in a variety of extracellular vesicles through the EVmiRNA website (http://bioinfo.life.hust.edu.cn/EVmiRNA#!). We speculated that EPCs-Exos modulated the expression pattern of miR-30e-5p in HUVECs by transferring miR-30e-5p. Therefore, EPCs was transfected with the miR-30e-5p inhibitor (*P* < 0.001, Figure 3(b)) and co-cultured with HUVECs. The partial recovery of miR-30e-5p by EPCs-Exos was reversed by EPCs-Exos^miR-inhibi^ (*P* < 0.001, Figure 3(a)). The ferroptosis-related index detection and cell behavior tests demonstrated that compared with the EPCs-Exos co-culture group, the content of GSH was reduced, lipid-ROS and MDA were significantly increased, the iron ion aggregation was increased, and the cell migration and angiogenesis were decreased in the EPCs-Exos^miR-inhibi^ co-culture group (all *P* < 0.001, Figure 3(c-h)). To further study whether miR-30e-5p existed in EPCs-Exos and EPCs-Exos regulated HUVEC injury by transferring miR-30e-5p, RNase protection assay was performed on EPCs-Exos to verify whether miR-30e-5p was encapsulated in Exos or existed on the surface of Exos (Figure 3(i)). After RNase treatment, the expression pattern of miR-30e-5p in the culture medium wasn’t changed, while the expression pattern was decreased after combination treatment with RNase and Triton 100, which indicated that miR-30e-5p was encapsulated in the membrane rather than directly released. These results suggested that EPCs-Exos regulated miR-30e-5p expression in HUVECs by transferring miR-30e-5p, thus improving ferroptosis and endothelial injury in HUVECs.

### miR-30e-5p targeted SP1

**Figure 4.**
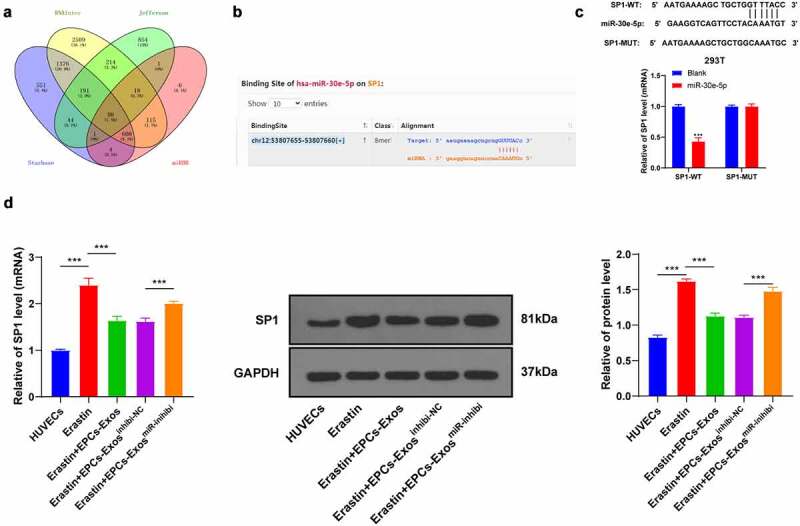
miR-30e-5p targeted SP1. (a) The downstream target genes of miR-30e-5p were predicted using the Starbase website (http://starbase.sysu.edu.cn), Jefferson website (https://cm.jefferson.edu/rna22/Precomputed), RNAInter website (http://www.rna-society.org/raid/search.html) and miRDB (http://mirdb.org); (b) The binding sites were predicted using the Starbase website; (c) The binding relationship was verified by dual-luciferase assay; (d) The expression of SP1 in HUVECs of each group was detected by RT-qPCR. The cell experiments were repeated 3 times independently. The data in the figure were all measurement data and were expressed as mean ± standard deviation. T test was used for data analysis in panel C and one-way ANOVA was used for data analysis in panel D. Tukey’s multiple comparisons test was used for the post hoc test. *** *P* < 0.001.

To further explore the regulatory mechanism of miR-30e-5p in HUVECs, the downstream target genes of miR-30e-5p were predicted using the Starbase website (http://starbase.sysu.edu.cn), Jefferson website (https://cm.jefferson.edu/rna22/Precomputed), RNAInter website (http://www.rna-society.org/raid/search.html) and miRDB (http://mirdb.org/) (Figure 4(a)), and SP1 was identified. SP1 plays an important role in HUVEC injury [21]. Therefore, we speculated that miR-30e-5p exerted effects on HUVECs through SP1. The binding sites between miR-30e-5p and SP1 were predicted using the Starbase website and the binding relationship was verified in 293 T cells by dual-luciferase assay (*P* < 0.001, Figure 4(b,c)). The mRNA and protein levels of SP1 in the HUVECs group, Erastin group, Erastin + EPCs-Exos group, Erastin + EPCs-Exos^inhibi-NC^ group, and Erastin + EPCs-Exos^miR-inhibi^ group were detected by RT-qPCR and WB (all *P* < 0.001, Figure 4(d)). The expression pattern of SP1 in HUVECs was upregulated after Erastin treatment and downregulated in HUVECs after adding with EPCs-Exos in the Erastin group, while EPCs-Exos^miR-inhibi^ partially reversed this trend (*P* < 0. 001). Overall, that miR-30e-5p targeted SP1.

### Overexpression of SP1 reversed the effect of EPCs-Exos on protecting HUVECs from ferroptosis and endothelial injury

**Figure 5.**
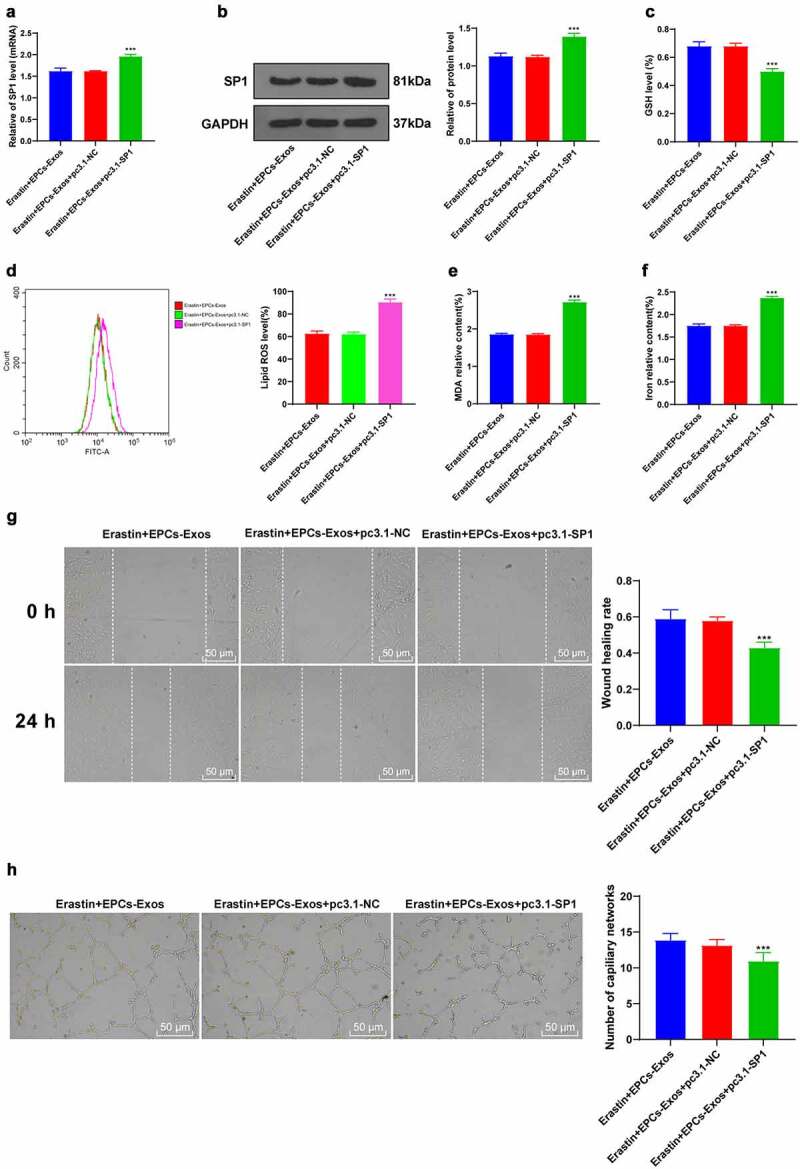
Overexpression of SP1 reversed the protective effect of EPCs-Exos on HUVECs against ferroptosis and endothelial injury. HUVECs were transfected with pcDNA3.1-SP1 and co-cultured with EPCs-Exos. The cell injury changes were observed. (a) The expression of SP1 was detected by RT-qPCR; (b) The protein level of SP1 was detected by WB; (c) GSH content determination; (d) Lipid-ROS content determination; (e) MDA content determination; (f) Iron ion content determination; (g) Cell migration was detected by scratch assay; (h) Cell angiogenesis detection; (i) The expression of miR-30e-5p was detected by RT-qPCR after Exos were treated with RNase and detergent. The cell experiments were repeated 3 times independently. The data in the figure were all measurement data and were expressed as mean ± standard deviation. One-way ANOVA was used for data analysis. Tukey’s multiple comparisons test was used for the post hoc test. *** *P* < 0.001. pc3.1-NC was pcDNA3.1-NC, pc3.1-SP1 was pcDNA3.1-SP1.

To verify the mechanism of SP1 on Erastin-induced HUVEC ferroptosis, a functional rescue experiment was carried out. HUVECs were transfected with pcDNA3.1-SP1 and co-cultured with EPCs-Exos and Erastin. The transfection efficiency of pcDNA3.1-SP1 was verified by RT-qPCR. The protein level of SP1 was determined by WB (Figure 5(a,b)). After upregulating the expression of SP1 in HUVECs again, GSH production was decreased, lipid-ROS and MDA productions were increased, and iron ion accumulation was increased (all *P* < 0.001, Figure 5(c-f)); cell migration and angiogenesis were decreased (all *P* < 0.001, Figure 5(g,h)). Collectively, these results suggested that overexpression of SP1 reversed the protective effect of EPCs-Exos on HUVECs against ferroptosis and endothelial injury.

### EPCs-Exos activated the AMPK pathway by miR-30e-5p targeting SP1

**Figure 6.**
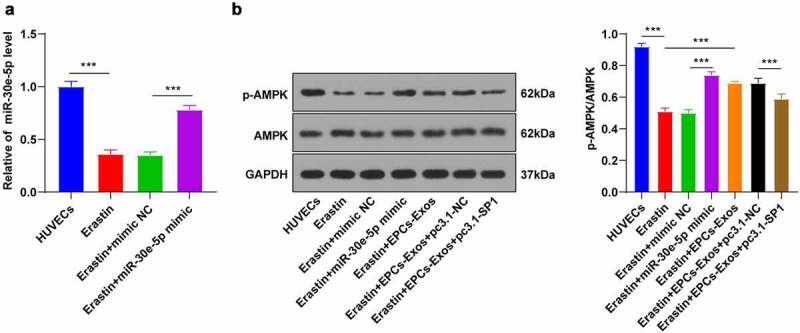
EPCs-Exos promoted the AMPK pathway activation through miR-30e-5p targeting SP1. In HUVECs treated with Erastin and transfected with miR-30e-5p mimic/NC, and HUVECs co-cultured with EPCs-Exos, treated with Erastin, and transfected with pcDNA3.1-SP1, the protein phosphorylation of the AMPK pathway was observed. A: The expression of miR-30e-5p was detected by RT-qPCR; B: The protein levels of p-AMPK and AMPK were detected by WB. The cell experiments were repeated 3 times independently. The data in the figure were all measurement data and were expressed as mean ± standard deviation. One-way ANOVA was used for data analysis. Tukey’s multiple comparisons test was used for the post hoc test. *** *P* < 0.001. pc3.1-NC was pcDNA3.1-NC, pc3.1-SP1 was pcDNA3.1-SP1.

SP1 can inhibit the AMPK pathway activation [22]. The AMPK pathway is closely associated with ferroptosis [23]. We speculated that EPCs-Exos might mediate the AMPK pathway and participate in Erastin-induced ferroptosis by regulating the expression pattern of SP1 in HUVECs. Firstly, the Erastin-treated HUVECs were transfected with miR-30e-5p mimic, with mimic NC as control. The transfection efficiency was detected by RT-qPCR (Figure 6(a)). The levels of AMPK protein and its phosphorylated protein in the HUVECs group, Erastin group, Erastin + mimic NC group, Erastin + miR-30e-5p mimic group, Erastin + EPCs-Exos group, Erastin + EPCs-Exos + pc3.1-NC group, and Erastin + EPCs-Exos + pc3.1-SP1 group were further detected by WB (Figure 6(b)). Compared with the HUVECs group, Erastin inhibited the phosphorylation of AMPK protein, while the AMPK phosphorylation was increased after transfection with miR-30e-5p mimic; compared with the Erastin group, the level of phosphorylation was increased in the EPCs-Exos group, while the promotive effect of EPCs-Exos co-culture on AMPK phosphorylation was inhibited again after transfection with pcDNA3.1-SP1 (all *P* < 0.001). Conjointly, miR-30e-5p carried by EPCs-Exos targeted SP1 expression in HUVECs treated with Erastin and activated the AMPK pathway, thus inhibiting ferroptosis and endothelial injury.

## Discussion

**Figure 7.**
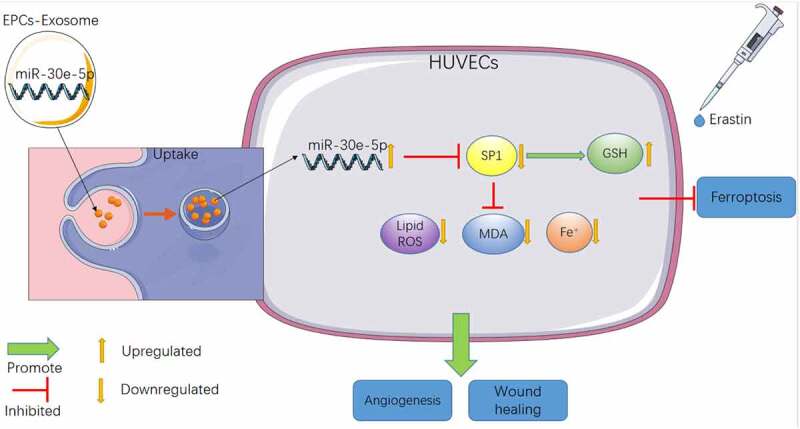
Mechanism diagram of EPCs-Exo uptake by HUVECs cells improving ferroptosis-induced by Erastin. HUVECs increased the level of intracellular GSH by inhibiting SP1 expression, reduced the levels of lipid ROS, MDA and iron ions, thus improving the decline of cell migration and angiogenesis caused by ferroptosis of HUVECs induced by Erastin.

There is ample evidence to suggest the participation of ferroptosis in numerous pathological processes including endothelial dysfunction, traumatic brain injury, cancer development, kidney injury, and neurodegenerative disease [6]. A previous study has demonstrated the involvement of Exos in ferroptosis regulation [10]. Our study illustrated that EPCs-Exos-shuttled miR-30e-5p regulated Erastin-induced ferroptosis in HUVECs via the SP1/AMPK axis (Figure 7).

GSH, Lipid-ROS, and MDA are typical ferroptosis-related indexes [24]. Our results demonstrated reduced GSH, increased lipid-ROS and MDA contents, and elevated iron ions in Erastin-induced HUVECs. Cell migration and angiogenesis are the main functions of HUVECs [25]. Our experimental results revealed that HUVEC migration and angiogenesis were decreased by Erastin induction, while the changes were partially improved after treatment with ferroptosis inhibitor Fer-1. Consistently, Erastin-induced ferroptosis impairs endothelial cell functions [5]. Collectively, Erastin-induced ferroptosis injured the endothelial functions of HUVECs.

A variety of miRNAs play a role in protecting against endothelial injury [26]. Our results demonstrated that miR-30e-5p expression in HUVECs was downregulated by Erastin induction, and partially recovered after the co-culture of EPCs-Exos with HUVECs. To explicate whether EPCs-Exos modulated miR-30e-5p expression in HUVECs by transferring miR-30e-5p, EPCs were transfected with miR-30e-5p inhibitor and co-cultured with HUVECs. Our results discovered that the partial recovery of miR-30e-5p by EPCs-Exos was reversed by EPCs-Exos^miR-inhibi^; GSH was reduced, lipid-ROS and MDA contents were enhanced, iron ion aggregation was increased, and cell migration and angiogenesis were repressed. Consistently, an existing study has documented the involvement of miR-30e-5p in the regulation of endothelial injury [14]. miR-30e-5p has been evidenced to regulate the expression of the uncoupling protein 2, a mitochondrial protein that decreases ROS formation by the mitochondria [27]. EPCs-Exos are of vital importance in protecting endothelial cells [9]. Furthermore, we identified that miR-30e-5p was encapsulated in the membrane of EPCs-Exos rather than directly released. In conclusion, EPCs-Exos modulated miR-30e-5p expression in HUVECs by transferring miR-30e-5p, thus improving ferroptosis and endothelial injury in HUVECs.

To study the mechanism of miR-30e-5p in HUVECs, we predicted the downstream target gene of miR-30e-5p and identified SP1. SP1 is a transcription factor that is found to exist in all cell types of mammals, which is known to be participated in the regulation of cell cycle, tissue-specific, and pathway response genes [28]. The binding sites of miR-30e-5p and SP1 were predicted using the Starbase website and the binding relationship was verified. Our results demonstrated that SP1 expression in HUVECs was upregulated by Erastin induction and downregulated after EPCs-Exos treatment, while EPCs-Exos^miR-inhibi^ induction partially reversed this trend. Consistently, SP1 modulates the injury of HUVECs [21]. In brief, miR-30e-5p targeted SP1. To verify the mechanism of SP1 in regulating Erastin-induced HUVECs ferroptosis, HUVECs were transfected with pcDNA3.1-SP1 and co-cultured with EPCs-Exos and Erastin. Our results elicited that after upregulating SP1 expression in HUVECs again, GSH production was reduced, lipid-ROS and MDA productions were increased, and iron ion accumulation was increased; cell migration and angiogenesis were repressed. Similarly, SP1 regulates GSH and MDA in human lens epithelial cells in diabetic cataracts [29]. Collectively, SP1 overexpression annulled the protecting effect of EPCs-Exos against HUVECs ferroptosis and endothelial injury.

An existing study identified the association between SP1 and the activation of the AMPK pathway [22]. The activation of AMPK has been documented to inhibit ferroptosis [23]. We speculated that EPCs-Exos might mediate the AMPK pathway and be involved in Erastin-induced ferroptosis by regulating SP1 expression in HUVECs. Our results noted that Erastin-inhibited AMPK protein and phosphorylation levels were increased by co-culture with EPCs-Exos, while the phosphorylation was inhibited again after transfection with pcDNA3.1-SP1. Energy-stress-mediated AMPK activation inhibits ferroptosis and AMPK deletion promoted ferroptosis induced by low-dose Erastin [23]. Consistently, previous findings have demonstrated that SP1 inhibits the activation of the AMPK pathway [22]. In conclusion, miR-30e-5p carried by EPCs-Exos targeted SP1 expression in HUVECs treated with Erastin and activated the AMPK pathway, thus inhibiting ferroptosis and endothelial injury.

In summary, the findings in this study supported that EPCs-Exos-shuttled miR-30e-5p regulated Erastin-induced ferroptosis in HUVECs via the SP1/AMPK axis. However, in this study, we only observed the regulatory mechanism of EPCs-Exos on the ferroptosis in HUVECs induced by Erastin *in vitro* and failed to verify it in animal models. In addition, there are a variety of miRNAs that are highly expressed in the Exos of endothelial cells and whether there are other molecular mechanisms involved in the regulation of endothelial cell ferroptosis remains to be studied. Further work is warranted to establish an animal model of endothelial cell ferroptosis and explore the molecular mechanism of EPCs-Exos on regulating endothelial cell ferroptosis and search for other molecular mechanisms of ferroptosis in endothelial cells.

### Conclusion

EPCs-Exos promoted the activation of the AMPK pathway by upregulating the expression of miR-30e-5p in HUVECs and inhibiting the expression of SP1 in HUVECs, thus inhibiting Erastin-induced cell ferroptosis.
